# Development and Quality Characteristics of Elderly-Friendly Pulpo a La Gallega Prepared Using Texture-Modified Octopus (*Octopus vulgaris*) Arms

**DOI:** 10.3390/foods12183343

**Published:** 2023-09-06

**Authors:** Sang-In Kang, Jin-Soo Kim, Sun-Young Park, Seok-Min Lee, Mi-Soon Jang, Jae-Young Oh, Jae-Suk Choi

**Affiliations:** 1 Seafood Research Center, IACF, Silla University, 606, Advanced Seafood Processing Complex, Wontang-ro, Amanam-dong, Seo-gu, Busan 49277, Republic of Korea; ftrnd5@silla.ac.kr; 2 Department of Seafood Science and Technology, Institute of Marine Industry, Gyeongsang National University, 2-9, Tongyeonghaean-ro, Tonyeong-si 53064, Gyeongsangnam-do, Republic of Korea; jinsukim@gnu.ac.kr (J.-S.K.); tjsdud3591@gnu.ac.kr (S.-Y.P.); tjrals03001@gnu.ac.kr (S.-M.L.); 3 Food Safety and Processing Research Division, National Institute of Fisheries Science, 216, Gijanghaean-ro, Gijang-eup, Busan 46083, Republic of Korea; suni1@korea.kr (M.-S.J.); ojy0724@korea.kr (J.-Y.O.)

**Keywords:** pulpo a La Gallega, elderly-friendly food, texture-modified technology, *Octopus vulgaris*, HMR (home meal replacement)

## Abstract

Considering the need for developing new senior-friendly processed seafood, this study aimed to develop octopus pulpo with high preference and excellent quality characteristics for elderly individuals by optimizing the vinegar immersion and sous vide softening treatment conditions for octopus (*Octopus vulgaris*) arms. The optimal sous vide heating temperature (70.0–100.0 °C), time (69.5–170.5 min), and vinegar concentration (0.2–0.8%) were established using response surface methodology (RSM). The pulpo prototype was produced using an octopus arm softened under optimal conditions and seasoned with a potato and olive oil sauce. The physicochemical and nutritional properties of the prototype were evaluated, followed by sensory evaluation and safety assessments. The hardness of softened octopuses obtained by 0.48% vinegar immersion and sous vide treatment (84.3 °C, 139.8 min), determined using RSM, was 394.5 × 1000 N/m^2^, showing a reduction of 83.0%; this was confirmed by electron microscopic observation. The texture of the pulpo prototype with softened octopus arms showed the highest preference (8.4 points) and high physicochemical and nutritional properties. Overall, the octopus pulpo a La Gallega prototype produced using texture-modified octopus arms was suitable for consumption by elderly people with chewing disorders and could help improve their quality of life.

## 1. Introduction

According to data from the National Statistical Office of the Republic of Korea, the aging population reached 7.0% in 2000, resulting in an aging society, and 14.2% in 2017, forming an aged society. By 2025, the elderly population is predicted to reach 20.6%, resulting in a super-aged society [1]. Further, the pace of aging (the number of years required to attain super-aging by an aging society) in Korea is very fast at 25 years, compared to 143 years in France, 88 years in the United States, 81 years in Italy, 77 years in Germany, and 35 years in Japan [1].

In general, elderly individuals suffer from eating disorders owing to the deterioration of their body structure, resulting in health problems. Four main types of eating disorders are noted in the elderly: (1) masticatory disorders caused by deterioration of functions related to chewing because of tooth loss; (2) dietary intake disorders caused by abnormal passage of food from the mouth to the stomach, i.e., dysphagia; (3) digestive disorders caused by a decrease in salivation, gastric and pancreatic digestive enzymes, and peristalsis; and (4) malnutrition caused by lack of food intake. Unlike overnutrition in the general working-age population (15–64 years old), nutritional disorders in the elderly are caused by the abnormal digestion of food resulting from eating disorders [2,3].

In Korea, standard specifications for senior-friendly foods are presented by the Ministry of Food and Drug Safety Food Code [4]) and the Korean Industrial Standards of the Ministry of Trade, Industry, and Energy [5]. Texture property standards (less than 500,000 N/m^2^) and nutritional standards (compliance with three or more of the nine nutritional standards presented) for elderly-friendly foods are specified in the Food Code. According to the Korean Industrial Standards, senior-friendly foods are classified into three levels based on their physical properties: Level 1 includes foods that can be eaten using teeth (less than 500,000–greater than 50,000 N/m^2^), Level 2 includes foods that can be eaten using gums (less than 50,000–more than 20,000 N/m^2^), and Level 3 includes foods that can be consumed using only the tongue (less than 20,000 N/m^2^). Further, satisfying at least one of the nine nutritional standards presented is necessary. Further, both the Korea Food Code and the Korean Industrial Standards indicate that all age-friendly foods need to meet the set standards for properties, condition, and hygiene.

Owing to the rapid increase in the number of elderly people, Korean companies are increasingly producing senior-friendly foods. In particular, the Ministry of Agriculture, Food, and Rural Affairs and the Ministry of Oceans and Fisheries announced 27 products from 8 companies on 29 October 2021, 26 products from 11 companies on 30 June 2022, and an additional five products on 30 September 2022. Further, 15 products from 15 companies were designated as senior-friendly foods of the best quality, which then increased to 79 products to date (Senior-Friendly Industry Support Center, Iksan, Jeollabuk-do, Korea; https://www.foodpolis.kr/seniorfood/bestPrd (accessed on 3 July 2023)). Senior-friendly foods are products that are manufactured and processed by adjusting their physical properties, shape, and ingredients to facilitate intake by elderly individuals, nutritional supplementation, digestion, and absorption, along with enhanced usability for the elderly.

Most of the 79 types of senior-friendly foods are processed agricultural products or livestock products, while only 8 products, including grilled flounder mousse (Shinsegae Foods, Inc., Seoul, Korea), rice porridge with cod and tofu and rice porridge with abalone and sea mustard (*Undaria pinnatifida*) (Pulmuone Foods Co. Ltd., Yongin, Chungcheongbuk-do, Korea), rice porridge with Hwang Tae (dried Alaska pollack meat) (Welfare Union Co., Ltd., Seoul, Republic of Korea), rice porridge with seafood (Green-Family Co., Ltd., Hwaseong, Gyeonggi-do, Republic of Korea), Stewed mackerel that can be eaten down to the softened bones (Hyundai Green Food Co. Ltd., Yongin, Gyeonggi-do, Republic of Korea), and egg rolls with katsuobushi (Ourhome Co., Ltd., Seoul, Republic of Korea), are related to seafood. Among these eight products, only one, i.e., grilled flounder mousse, has seafood as the main ingredient. However, to the best of our knowledge, senior-friendly food products containing octopus have not yet been developed.

Technologies for developing elderly-friendly foods result in the improvement of physical property, nutrient content, hygiene, and digestibility. Physical property improvement technologies for senior-friendly food include physical processes (high-temperature and high-pressure treatment, superheated steam roasting, heat treatment using boiling water and steaming, ultra-high-pressure treatment, grinding, and cutting), chemical processes (pH adjustment, treatment with phosphate and sodium bicarbonate, etc.), and biological processes (fermentation and enzymatic hydrolysis). Nutrient enrichment techniques include the addition of food additives such as calcium chloride, potassium chloride, calcium carbonate, ascorbic acid, and other vitamins. Hygiene enhancement technologies include high-temperature and high-pressure treatment, ultra-high-pressure treatment, pH control, and preservative treatment. Digestibility improvement technologies include various processing treatments, such as high-pressure heating, superheated steam roasting, ultra-high-pressure, grinding and cutting, boiling in water or steaming, fermentation, and enzymatic hydrolysis [6,7].

Sous vide, meaning ‘under vacuum’ in French, refers to a process wherein ingredients are placed in a sanitary plastic bag, sealed in a vacuum, and cooked for a long time in a water bath at 54–90 °C. The purpose of sous vide is to evenly heat the outside and inside of food and maintain moisture in food [8]. Sous vide treatment has been reported to alter the tissue physical properties of various aquatic products, including Atlantic mackerel fillets (*Scomber scombrus*), spotted seerfish steak (*Scomberomorus guttatus*), sturgeon fillets (*Acipenser gueldenstaedti*), lobster (*Homarus americanus*), and Indian white shrimp (*Fenneropenaeus indicus*) [9], as well as in mollusks, such as octopus (*Octopus vulgaris*), squid (*Loligo vulgaris*), and cuttlefish (*Sepia officinalis*) [10].

Among aquatic products, cephalopods, such as octopus and squid, belonging to the phylum Mollusca, require heating to a degree at which the texture (physical property) is soft for consumption. However, excess heating results in a tough texture; therefore, they must be heated to an extent at which there is no significant change in texture and taste [10,11]. In particular, to obtain a high preference for consumers with weak chewing ability or elderly consumers, a product manufactured using the appropriate heat-treatment conditions is needed.

Further, consumers are demanding various products that ensure convenience of food intake, health, and nutrition while undergoing lifestyle [12,13]. Therefore, as an alternative to expanding the current range of consumer food choices, convenient foods, including various home meal replacements (HMRs) and meal kits with health and nutritional components, are being developed and sold [14]. Although a unique dietary culture has existed in Korea since the past, the Westernized food culture is gradually increasing [15], and many foods from other countries are being enjoyed by Koreans [16]. For example, pizza and pasta from Italy [17], as well as gambas al ajillo and pulpo a La Gallega (polbo á feir) from Spain [18], are consumed by various age groups. Considering some of the most popular foods worldwide, it is now necessary to develop new processed seafood preparations that meet the preferences of elderly consumers. On the other hand, home meal replacement using webfoot octopus and squid [19,20] and elderly-friendly processed seafood products using pollack, halibut, mackerel, seaweed, and abalone have been developed. However, no studies have been conducted on the development of products such as convenience food or senior-friendly food using octopus.

Therefore, in this study, by improving the texture of octopus and establishing a flavor improvement process, we developed an elderly-friendly octopus pulpo a La Gallega formulation using an octopus (*Octopus vulgaris*) arm whose texture was modified through sous vide treatment, and its quality characteristics were evaluated.

## 2. Materials and Methods

### 2.1. Materials

Five fresh common octopus (*Octopus vulgaris Cuvier*) with body weight (572.0–641.4 g (606.7 ± 34.7 g)) and body length (22.5–28.3 cm (25.4 ± 2.9 cm)) used in the experiment were purchased from Samsamhaemul Co., Ltd. (Geoje-si, Gyeongsangnam-do, Republic of Korea) in November 2022. To prepare octopus pulpo a La Gallega, flour (Daehan Flour Mills Co., Ltd., Seoul, Republic of Korea), olive oil (Sajo Daerim Co., Ltd., Seoul, Republic of Korea), sugar (CJ Cheiljedang Corp., Seoul, Republic of Korea), paprika powder (IF&S Food Co., Ltd., Osan, GyeonggI-do, Republic of Korea), parsley powder (Shinyoung FS Co., Ltd., Gwangju, GyeonggI-do, Republic of Korea), black pepper (Ottogi Co. Ltd., Anyang, GyeonggI-do, Republic of Korea), vinegar (Ottogi Co., Ltd., Hanam, GyeonggI-do, Republic of Korea), salt (Hanju, Ulsan, Republic of Korea), and fresh potatoes were purchased from a large consumer mart located in Tongyeong (Gyeongsangnam-do, Republic of Korea). All reagents used in the experiments were of analytical grade.

### 2.2. Manufacture of Octopus Pulpo a La Gallega Prototypes

To soften octopus arms (17.5 ± 0.8 cm), 756 g of fresh octopus arms and 0.2, 0.3, 0.5, 0.7, and 0.8% vinegar were added to a vacuum-packed plastic bag (Nylon/PE, 15 × 24 cm, 80 μm, Intrise Co. Ltd., Ansan, Gyeonggi-do, Republic of Korea), and sous vide treatment was performed for 69.5, 90.0, 120.0, 150.0, and 170.5 min at temperatures of 70.0, 76.0, 85.0, 94.0, and 100.0 °C using a sous vide device (SV-8008, Biolomix Co. Ltd., Wilmington, NC, USA). A prototype octopus pulpo a La Gallega dumpling was prepared by mixing 76.0% (*w*/*w*) of a softened octopus arm, 190 g of cooked (100 °C, 20 min) potatoes (2 × 2 × 2 cm), and seasoning sauce in a packaging container (polyethylene terephthalate; 20 × 12 × 6 cm; Goodfellow Co., Ltd., Huntingdon, UK). The seasoning sauce was prepared by mixing 23 g of salt, 18 g of paprika powder, 7 g of sugar, 1.5 g of salt, 0.4 g of pepper, and 0.1 g of parsley (Table 1).

### 2.3. Establishment of Octopus Sous Vide Conditions by Response Surface Methodology (RSM)

For predicting and confirming the optimal temperature (°C) and time (min) of sous vide treatment and the acetic acid concentration (%) for manufacturing octopus pulpo prototypes, MINITAB statistical program (MINITAB Ver. 18; MINITAB LLC., State College, PA, USA) was used. The correlation between the independent and dependent variables was confirmed using MAPLE software (MAPLE Ver. 12, Waterloo Maple Inc., Waterloo, ON, Canada). After setting the temperature and time of the sous vide treatment and the acetic acid concentration for octopus softening as independent variables, the treatment temperature and time, as well as the acetic acid concentration ranges to be entered into the central composite design (CCD) of RSM, were set (Table 2). Subsequently, referring to the minimum and maximum values determined through preliminary experiments, *X_1_* (sous vide treatment temperature), *X_2_* (sous vide treatment time), and *X*_3_ (acetic acid concentration) for application to the central synthesis plan were coded in 5 steps, and as mentioned in Table 3, 17 types of octopus pulpo prototypes were randomly manufactured and the experiment was performed. The dependent variables of the manufacturing conditions of the octopus pulpo prototype at this time were hardness (*Y*_1_), total acidity (*Y*_2_), and sensory texture (*Y*_3_) through descriptive analysis. These data were further used for regression analysis.

### 2.4. Hardness

Using “Experiment methods 1” according to the method described in the Korean Industrial Standards [5] and the Food Code [4], as well as “Experiment methods 3” according to the Korean Industrial Standards (Figure S1) [5], the hardness of test materials was measured using a texture analyzer (CT3-1000, Brookfield, Middleboro, MA, USA).

### 2.5. Total Acidity Analysis

Total acidity was measured using the method described by Vanderzant and Splittstoesser [21]. Briefly, 90 mL of distilled water was added to 10 mL of the sample and mixed; NaOH (0.1 N) was added to the mixture and titrated until the pH reached 8.30. The required amount of NaOH was expressed in terms of lactic acid according to the following formula:

$$Acidity\;(\%)=\frac{mL\;of\;0.1N-NaOH\times 0.1N-NaOH\;factor\times 0.0090\times dilution\;factor}{Sample\;amount\;(g)}\times 100$$

### 2.6. Volatile Basic Nitrogen (VBN) Analysis

The volatile base nitrogen content was measured by the micro-diffusion method using Conway’s units [22]. Tertiary distilled water (50 mL) was added to 10 g of the sample, stirred, leached for 30 min, and filtered to prepare a sample solution. One milliliter of the sample solution and 1 mL of saturated K_2_CO_3_ were then placed in the outer cell of the Conway diffusion unit, and 1 mL of 0.01 N H_2_SO_4_ and 2–3 drops of the indicator was added to the inner cell of the Conway unit. The reaction was then performed at 37 °C for 120 min while the Conway unit was sealed with a glycerin-coated lid. The volatile base nitrogen content was titrated with 0.01 N sodium hydroxide to the inner cell of the Conway unit and then calculated using the following formula:

$$VBN\;(mg/100g)=\frac{(sample\;titration\;value-control\;titration\;value)\times 0.14\times factor\;of\;0.01N\;NaOH\times dilution\;rate}{Sample\;amount\left(g\right)}\times 100$$

### 2.7. Total Bacterial Count (TBC) Analysis

Total bacterial counts (TBCs) were analyzed following the AOAC [23] guidelines. A specific quantity of the sample (25 g) was collected for TBC measurement, and 225 mL of 0.85% sterile saline was added. The mixture was homogenized using a stomacher (Bag Mizer 400 VW, Interscience, France) for 30 s, and the resulting homogeneous solution was gradually diluted. Subsequently, for TBC determination, the preprocessed sample and surface sample solution were inoculated onto PCA medium (Difco^TM^ plate count agar, Becton, Dickinson, and Company, Franklin Lakes, NJ, USA), cultured at 35 ± 1 °C for 48 h, and the colony count was recorded.

### 2.8. Determination of Proximate Components (Moisture, Protein, Fat, Ash, and Carbohydrates) and Calories

For determining proximate components, approximately 0.5 g of ground sample was analyzed using the atmospheric pressure drying method for moisture, the semi-micro Kjeldahl method for crude protein, the Soxhlet method for crude fat, and the dry ashing method for ash, according to the Food Code [4]. Carbohydrates were calculated as 100 − (moisture content + crude protein content + crude fat content + ash content). The amount of carbohydrates was the sum of the crude fiber and sugar. Calories were calculated based on the general component analysis data but applied to fish and shellfish among the energy conversion coefficients of the Japanese Food Composition Table [24].

### 2.9. Salinity Analysis

Salinity was measured using the dry-ashing method described in the Food Code [4]. After incinerating an amount of sample containing approximately 1 g of table salt and dissolving it in water, 2–3 drops of potassium chromate (K_2_CrO_4_) solution were added to 10 mL of the rectified solution (500 mL) and filtered filtrate, followed by adding 0.02 N silver nitrate (AgNO_3_) for titration. Salinity was calculated using the following formula:

$$Salinity\left(\%\right)=\frac{A\times F\times 5.85}{\begin{array}{c} \mathrm{Sample}\;\mathrm{amount}\;\left(g\right) \end{array}}$$

A:0.02 N silver nitrate solution (mL) consumed for titration; F:factor of 0.02 N silver nitrate solution

### 2.10. Total Amino Acid Analysis

Samples for the total amino acid analysis were pretreated as described in the Food Code [4]. To prepare pretreatment samples for analysis, a certain amount of sample and 10 mL of 6 N hydrochloric acid containing 0.05% (*v*/*v*) 2-mercaptoethanol were added to a test tube for hydrolysis. After sealing the test tube for hydrolysis, heat treatment (100 ± 1 °C, 24 h) was performed in a heating block (HF21; Yamato Scientific Co., Tokyo, Japan). Samples for amino acid analysis were prepared by cutting the sealed tube, concentrating and drying the hydrolyzed sample at 40 °C under reduced pressure to remove hydrochloric acid, and finally diluting (25 mL) the dried sample with 0.2 N sodium citrate buffer (pH 2.2). Total amino acids were analyzed in the pretreated sample using an amino acid automatic analyzer (Pharmacia Biotech Biochrom 30; Biochrom Ltd., London, UK), followed by identification and calculation.

### 2.11. Fatty Acid Analysis

The sample oil for the fatty acid composition analysis was prepared by extracting the sample to be analyzed as described by Bligh and Dyer [25], using a mixture of chloroform-methanol (2:1, *v*/*v*) as the extraction solvent. The internal standard used to calculate fatty acid content was methyl tricosanoate (99%; Sigma-Aldrich Korea, Seoul, Republic of Korea). Methyl tricosanoate (0.01 g) was dissolved in a chloroform solution to make 10 mL and a final concentration of 1 mg/mL. The fatty acid analysis produced fatty acid methyl ester derivatives according to the experimental method of the Food Code [4] using the extracted sample oil. Thereafter, fatty acid methyl ester derivatives were analyzed using gas chromatography (Shimadzu 14A, Shimadzu Co., Kyoto, Japan) equipped with a capillary column (Supelcowax-10 fused silica wall-coated open tubular column, 30 m × 0.25 mm I.d.; Supelco Japan Ltd., Tokyo, Japan). The temperature of the gas chromatography injector and detector (flame ionization detector) was set to 250 °C each; the column temperature was raised to 230 °C and maintained for 15 min. The carrier gas used was He (1.5 kg/cm^2^), and the split ratio was 1:50. Fatty acids were identified by comparing the retention time and equivalent chain length of the sample to those of the standard solution (Supelco 37 Component FAME Mix, Sigma-Aldrich Korea) analyzed under the same conditions as the sample [26].

### 2.12. Mineral Analysis

The minerals contained in the samples were analyzed as described in the Food Code [4]. For the analysis, 1 g of the sample was pretreated using a wet method under high temperature and reduced pressure conditions (80 ± 5 °C, 400 min). Inorganic analyses of the pretreated samples were performed using inductively coupled plasma mass spectrometry (ICP-MS, X Series II; Thermo Fisher Scientific, Waltham, MA, USA).

### 2.13. Analysis of Lead and Cadmium

Sample pretreatment for Pb and Cd analysis was performed according to the Food Code [4]. Analysis of Pb and Cd in the pretreated samples was performed using an inductively coupled plasma spectrophotometer (ICP; Atomscan 25, Thermo Fisher Scientific Inc., Waltham, MA, USA).

### 2.14. Sensory Texture for Optimizing the Manufacturing Conditions of the Octopus Pulpo a La Gallega

Descriptive analysis by a trained panel was approved by the Institutional Review Board (IRB) for human subject researchers (No. GIRB-G22-Y-0058) by the Bioethics and Safety Act. It comprised five members (20 s–30 s, 3 males, 2 females) trained in the characteristics of age-friendly foods; upon descriptive analysis, the sensory texture of octopus pulpo a La Gallega was assigned the highest score of 7 points (“very like”), an average score of 4 points (“normal preference”), and a lowest possible score of 1 point (“very dislike”). Also, any sample less than a score of 4 was considered unacceptable.

### 2.15. Preference Evaluation of Prototypes

A preference evaluation of the developed octopus pulpo prototype was conducted for the general elderly as follows: After obtaining approval (No. GIRB-G22-Y-0058) for research using human subjects from the Institutional Review Board (IRB; Jinju, Republic of Korea) of Gyeongsang National University (Jinju, Republic of Korea) in accordance with the Enforcement Decree of Bioethics and Safety Act, a sensory evaluation was conducted on the octopus pulpo a La Gallega prototype. Preference evaluation was conducted using a hedonic scale on 30 participants (15 males and 15 females; age distribution, 65–75 years; average age, 70 years) living at Tongyeong Senior Care Center (Tongyeong-si, Republic of Korea). The taste, texture, color, smell, and overall acceptance of the prototype were evaluated according to Lawless and Heymann [27].

### 2.16. Scanning Electron Microscope (SEM)

The microstructure of octopus arm muscle tissue was observed using a scanning electron microscope as described by Rattanasatheirn et al. [28]. After cutting the muscle of the octopus arm into a regular shape (4 mm × 4 mm × 4 mm), it was immersed in 2.5% glutaraldehyde with 0.2 M phosphate buffer (pH 7.2; as a solvent) at 4 ± 1 °C for 24 h. Thereafter, these samples were washed thrice with deionized water for 15 min and then dehydrated for 30 min each in 20%, 40%, 60%, 80%, and 100% ethanol for a total of 150 min. These dehydrated octopus arm muscles were dried (dried at 10 °C for 10 min, then at 32 °C for 10 min) using a critical point dryer (13200-AB, SPI SUPPLIES, West Chester, PA, USA). The dried octopus muscle was coated with 100% gold (nanopowder, 200 nm particle size (SEM), 99.9% (metals basis), Sigma-Aldrich Co. Ltd., St. Louis, MO, USA) using an ion sputter coater (Sputter Coater SPT-20, Coxem Co., Republic of Korea). The microstructure of the gold-coated octopus arm muscles was observed at 10,000× magnification using a scanning electron microscope (SEM; JSM-7610F; Jeol Ltd., Tokyo, Japan).

### 2.17. Statistical Analysis

For the standard deviation and significant difference test (5% significance level) of the data for the results of these experiments (hardness, total acidity, acceptability evaluation, proximate composition, salinity, mineral, pH, VBN, heavy metals, and digestibility), analysis of variance (ANOVA) was performed using SPSS statistical package (SPSS for Windows, release 10.1), and Duncan’s multiple range test was performed after ANOVA.

## 3. Results

### 3.1. Effects of Sous Vide and Vinegar Treatment Conditions on Octopus Arm Hardness

A total of 27 samples were prepared by coding each of these variables in 5 stages (Table 1) to optimize the sous vide treatment temperature (°C), sous vide treatment time (min), and vinegar concentration (%) required for softening the octopus arm. Table 2 lists the hardness values (*Y*_1_) of the 27 samples. When examining the correlation between the independent and dependent variables in the response model equation using ANOVA, the *p*-value of the lack of fit test, which indicates the suitability of the response surface model, was 0.269, which was higher than 0.05, and the *p*-value of the model was 0.000, which was lower than 0.05 (Table 3). As the coefficient of determination (R^2^) was 0.988, which is close to 1 (Table 4), the RSM design model was considered suitable [29]. In the lack of fit test, a *p*-value higher than 0.05 can be regarded to indicate a suitable model [30,31].

**Figure 1:**
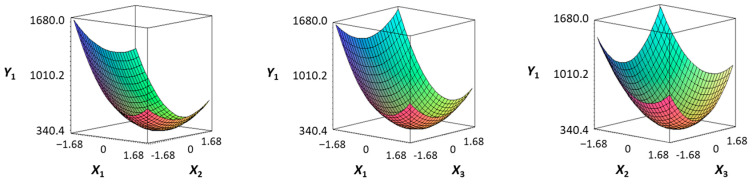

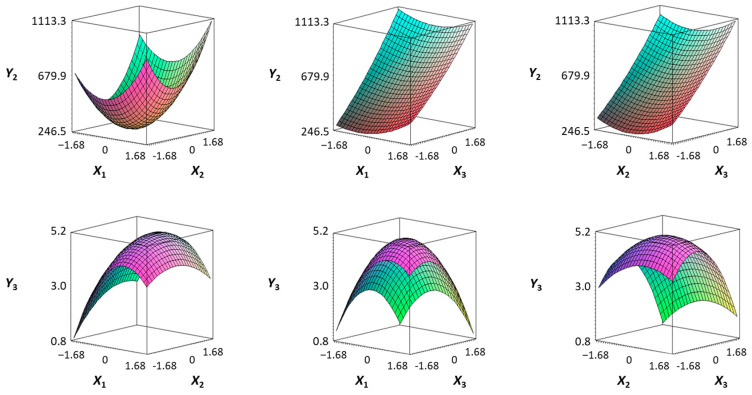
Three-dimensional response surface plots for preparing restructured pulpo a La Gallega from octopus arm based on the hardness (*Y*_1_), total acidity (*Y*_2_), and sensory texture (*Y*_3_).

The hardness (*Y*_1_) of the octopus arm according to the change in the sous vide treatment temperature (*X*_1_) decreased rapidly as the code value of *X*_1_ moved from −1.414 to +0.55. On the contrary, in the change of *X*_1_ from +0.55 to +1.414, the hardness of the octopus arm increased rapidly. In the case of sous vide treatment time (*X*_2_) and vinegar concentration (*X*_3_), as the code value moved from −1.414 to +0.61 and −0.20, respectively, the hardness of the octopus arm decreased slightly and reached a minimum. Until the two types of code values reached +1.414, the hardness of the octopus arm showed an increasing tendency (Table 5, Figure 1).

The results of this experiment confirmed that the hardness change of the octopus arm was the most affected by the sous vide treatment temperature among the sous vide treatment and vinegar immersion conditions [32,33].

### 3.2. Effects of Sous Vide Treatment and Vinegar Immersion Conditions on the Total Acidity of Octopus Arms

The results of the total acidity (*Y*_2_) of the 27 samples prepared according to the design of the RSM are shown in Table 2. When the correlation between the independent variable and the dependent variable of the response model equation was confirmed using ANOVA, the *p*-value of the lack of fit test, which indicates the suitability of the response surface model, was 0.470, higher than 0.05, and the *p*-value of the model was 0.000, lower than 0.05, (Table 4). The coefficient of determination (R^2^) was 0.996, which was close to 1; therefore, the model of this RSM design was considered suitable [29]. The total acidity (*Y*_2_) decreased slightly as the code value moved from −1.414 to −0.32 (*X*_1_) and −0.21 in the case of X_1_ (sous vide treatment temperature) and *X*_2_ (sous vide treatment time), respectively. Additionally, the total acidity increased slightly until the code values of *X*_1_ and *X*_2_ reached +1.414.

In the case of *X*_3_ (acetic acid concentration), the total acidity increased significantly as the code value increased from −1.414 to +1.414 (Table 5, Figure 1).

The results of this experiment, when the sous vide treatment and vinegar immersion conditions were different, confirmed that the vinegar immersion concentration had the greatest effect on the change in the total acidity of the octopus arm.

### 3.3. Effects of the Sous Vide Treatment and Vinegar Immersion Conditions on the Sensory Texture of Octopus Arms

The results of the sense texture (*Y*_3_) of 27 samples prepared according to the design of the RSM are shown in Table 2. When the correlation between the independent and dependent variables of the response model equation was confirmed using ANOVA, the *p*-value of the lack of fit test, which indicates the suitability of the response surface model, was 0.131, higher than 0.05; the *p*-value of the model was 0.000, lower than 0.05 (Table 3); and the coefficient of determination (R^2^) was 0.965, which was close to 1. Thus, the model of this RSM design was considered suitable [29].

In terms of the sensory texture (*Y*_3_) of the octopus arm, for *X*_1_ (sous vide treatment temperature), the sensory texture increased rapidly as the code value moved from −1.414 to 0.49. From the subsequent code value of +1.414, the sensory texture decreased rapidly. For *X*_2_ (sous vide processing time), the sensory texture of the octopus arm increased slightly from the code value of −1.414 to 0.22. From the subsequent code value of +1.414, the sensory texture of the octopus arm decreased slightly. For *X*_3_ (acetic acid concentration), the sensory texture of the octopus arm increased rapidly as the code value moved from −1.414 to −0.56. From the subsequent code value of +1.414, the sensory texture of the octopus arm decreased rapidly (Table 5, Figure 1).

Based on these results, the change in the sensory texture of the octopus arm, depending on the sous vide treatment and vinegar immersion conditions, was confirmed to be the most affected by the sous vide treatment temperature and vinegar concentration.

### 3.4. Optimization of Vinegar Immersion and Sous Vide Softening Treatment Conditions for Raw Octopus Arms

Table 6 shows the results of verifying the significance of the predicted and experimental values of hardness (*Y*_1_), total acidity (*Y*_2_), and texture preference (*Y*_3_) of raw octopus arm depending on the sous vide treatment temperature (*X*_1_), sous vide treatment time (*X*_2_), and vinegar immersion concentration (*X*_3_) using the RSM statistical technique.

The hardness of the softened octopus arm, according to the optimal conditions (*X*_1_, *X*_2_, and *X*_3_) predicted by the RSM program was 405.0 × 1000 N/m^2^, total acidity was 550.7 mg/100 g, and sensory texture was 4.8 points. The actual measured values of the octopus arm prepared by softening under optimal conditions were 394.5 × 1000 N/m^2^ for hardness, 551.0 mg/100 g for total acidity, and 4.9 points for sensory texture, showing no significant difference between the predicted value and the actual measured value (*p* > 0.05). Therefore, the optimal sous vide softening treatment temperature for the octopus arm was 84.3 °C, the sous vide softening treatment time was 139.8 min, and the vinegar immersion concentration was 0.48%.

### 3.5. Proximate Composition, Energy, and Salinity

Table 7 shows the general components, energy, and salinity of the common octopus used as raw octopus arm and the pulpo a La Gallega prototypes. The contents of general components per 100 g of raw octopus arm were 82.0 g water, 15.5 g crude protein, 0.6 g crude fat, 1.7 g ash, and 0.2 g carbohydrates. The contents of general components per 100 g of octopus pulpo a La Gallega were 69.6 g water, 16.9 g crude protein, 7.9 g crude fat, 1.8 g ash, and 3.8 g carbohydrates.

Both the raw octopus arm and prototype were considered foods with crude protein as the main component, except for water. Except for water, the general components of mollusk meat mainly comprise proteins; further, the water content is higher than that of fish whereas the protein content is low [34].

The caloric value calculated based on the content of general ingredients per 100 g of raw octopus arm was 72.4 kcal, which was slightly higher than the energy value of 68.0 kcal in octopus as described by the National Institute of Fisheries Science [35]. This was considered a result of differences in the harvest time, region, and size of the octopus. The caloric value per 100 g of the octopus pulpo a La Gallega prototype was 142.4 kcal, which accounted for 7.1% and 8.9% of the estimated energy requirements in males and females, respectively (2000 kcal for men and 1600 kcal for women) [36], in the age range of 65–74 years.

The salinity per 100 g of raw octopus arm was 0.6 g, which corresponds to 18.2% of the daily salt intake (3.3 g for men and women) for men and women of elderly age (65–74 years) [36]. The salt content per 100 g of the octopus pulpo a La Gallega prototype was 0.6 g, which was 18.2% of the daily salt intake of men and women aged 65–74 years (3.3 g for men and women) [36]. The octopus pulpo a La Gallega prototype was found to contain low levels of sodium when consumed by older adults.

### 3.6. Total Amino Acid Content

The total amino acid content of raw octopus arm and OPLGP is shown in Table 8. The total amino acid content per 100 g of raw octopus arm was 14,857.5 mg, and the main amino acids were glutamic acid (2384.3 mg, 16.0%) and aspartic acid (1576.1 mg, 10.6%). The essential amino acid content per 100 g of raw octopus arm was 7263.8 mg, which constituted approximately 48.9% of the total amino acids. The first limiting amino acid in the raw octopus arm was methionine (175.1 mg, 1.2%). However, the contents of threonine and lysine, which are the limiting amino acids of cereals, per 100 g of raw octopus arm were also high at 706.2 mg (4.7%) and 1196.6 mg (8.1%), respectively.

The total amino acid content per 100 g of the OPLGP was 15,632.4 mg. In 100 g of the OPLGP, the main amino acids (composition ratio greater than 8%) were leucine (1275.5 mg, 8.2%), aspartic acid (1737.7 mg, 11.1%), and glutamic acid (2500.8 mg, 16.0%), respectively. The total content of essential amino acids per 100 g of the OPLGP was 7325.3 mg, which was less than half (46.8%) of the total amino acid content. However, the content of threonine and lysine, which are the limiting amino acids in cereals was 772.9 mg (4.9%) and 1221.5 mg (7.8%), respectively. Therefore, if the elderly consume an appropriate amount of OPLGP as a meal replacement, they will be able to consume balanced nutrients.

### 3.7. Mineral Content

The mineral content per 100 g of raw octopus arm and OPLGP, divided into major and trace minerals, is presented in Table 9. The trace mineral content per 100 g of raw octopus arm was 26.2 mg for calcium, 305.0 mg for potassium, 3.5 mg for iron, and 0.8 mg for zinc, respectively, which corresponded to 38.9% and 8.9%, respectively, of the recommended daily intake (iron and zinc, both 9 mg) in the elderly (65–74 years old) among the nutritional intake standards for Koreans presented by the Ministry of Health and Welfare.

The mineral contents per 100 g of the OPLGP were 21.7 mg for calcium, 170.6 mg for potassium, 5.7 mg for iron, and 0.4 mg for zinc (Table 9). Compared to the mineral standards (calcium: Food Code [4] 70 mg or more, Korean Industrial Standard [5] 80 mg or more; potassium: all 350 mg or more) for senior-friendly foods presented by the Food Code [4] and Korean Industrial Standards [5], the calcium content was higher, but the potassium content was lower.

### 3.8. Fatty Acid Contents

The fatty acid contents of raw octopus arm and OPLGP are shown in Table 10. The total fatty acid content per 100 g of raw octopus arm was 289.4 mg. Polyunsaturated fatty acids were the highest at 144.6 mg (50.0%), accounting for half of the total fatty acids. These were followed by saturated fatty acids (93.8 mg, 32.6%) and monounsaturated fatty acids (51.0 mg, 17.5%) (Table 10). The main fatty acids per 100 g of raw octopus arm were saturated fatty acids 16:0 (48.2 mg, 16.7%), monounsaturated fatty acids 18:1n-9 (31.1 mg, 10.7%), polyunsaturated fatty acids, and omega-3 fatty acids 20:5n-3 (eicosapentaenoic acid, EPA) (54.2 mg, 18.7%) and 22:6n-3 (docosahexaenoic acid, DHA) (79.7 mg, 27.5%). These results confirm that the fatty acid composition of raw octopus arm is consistent with that of typical aquatic products [37,38].

The total fatty acid content per 100 g of the OPLGP was 6305.1 mg, which corresponded to 79.8% of the lipid content in the prototype (Table 10). Among the fatty acid content and lipid composition of the OPLGP, monounsaturated fatty acid had the highest content at 3948.8 mg (62.6%), followed by saturated fatty acids (1244.8 mg, 19.7%), and polyunsaturated fatty acids had the lowest content at 1111.5 mg (17.6%). The major fatty acids were 18:1n-9 (3822.6 mg, 60.2%), 16:0 (856.8 mg and 13.5%), and 18:2n-6 (665.1 mg, 10.5%). Lipids from the OPLGP exhibit a fatty acid composition pattern typical of olive oil [39].

### 3.9. pH and Volatile Basic Nitrogen (VBN)

Table 11 shows the results of pH and VBN as freshness indicators for raw octopus arm and OPLGP. The raw octopus arm had a pH of 6.70 and a volatile base nitrogen content of 6.5 mg/100 g, which was very fresh. In general, as an indicator of aquatic products, pH is classified as very fresh at 6.3 or higher, fresh at 5.9–6.3, poor in freshness at 5.2–5.9, and spoiled at less than 5.2. Volatile basic nitrogen content was judged as fresh when in the range of 5–10 mg per 100 g of fish, normal freshness in the range of 15–25 mg, early decay if in the range of 30–40 mg, and spoiled if greater than 50 mg [34]. The pH of the OPLGP was 6.92, and the volatile basic nitrogen content was 14.8 mg/100 g, which was 127.6% higher than that of raw octopus arm.

### 3.10. Total Bacterial Counts (TBC)

Table 11 shows the total bacterial counts of raw octopus arm and OPLGP. The total bacterial count of raw octopus arm was 2.0 × 10 CFU/g, which was very fresh. In general, total bacterial concentrations of less than 10^5^ CFU/g of the marine product are considered fresh; early spoilage is considered in the range of more than 10^5^ to less than 10^6^ CFU/g, and spoilage is considered at more than 1.5 × 10^6^ CFU/g [40,41]. The total bacterial count of the OPLGP was 1.2 × 10 CFU/g.

### 3.11. Heavy Metal Contents

According to the Korean Food Code [4], the permissible concentration of heavy metals in mollusks (shellfish and mollusks) is suggested to be less than 2.0 mg/kg for both lead and cadmium. Table 11 shows the heavy metal content of raw octopus arm and OPLGP. The heavy metal content of the octopus was detected as 0.06 mg/kg for lead and 0.09 mg/kg for cadmium, which corresponded to 3.0% of lead and 4.5% of cadmium compared to the standards presented in the Food Code. Therefore, it was considered safe in terms of heavy metal content. The heavy metal content of the OPLGP was 0.01 mg/kg for lead and 0.02 mg/kg for cadmium.

### 3.12. Scanning Electron Microscopy (SEM) Observations

**Figure 2:**
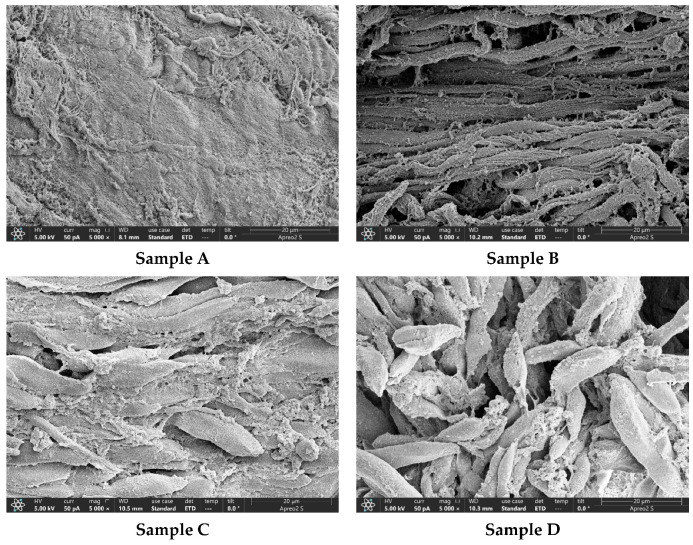
Scanning electron microscopy images (magnification: ×5000) of octopus arm ((**A**) raw octopus arm; (**B**) boiled octopus arm; (**C**) sous vide octopus arm; (**D**) soaked in vinegar and sous vide octopus arm).

Figure 2 shows the microstructure of raw octopus arm muscle tissue and the octopus pulpo, a La Gallega prototype. In general, the microstructure of seafood muscle exhibits different textures depending on the type of heat treatment [42,43]. The microstructure (A) of raw octopus arm without any heat treatment showed dense muscle fiber tissue without protein denaturation; however, when examining the microstructure (B) of the boiled octopus arm, the fascia constituting the muscle fiber was found to be cracked, and the muscle fiber tissue gap was widened.

When examining the microstructure of octopus arm muscles (C) treated only with sous vide treatment (84.3 °C, 139.8 min) without vinegar treatment, the gap between the muscle fibers of Sample C, compared to that in the microstructure of Samples A and B. The gap between the muscle fiber tissues in the microstructure (D) of octopus arm muscles after immersion in 0.5% vinegar solution and sous vide treatment (84.3 °C, 139.8 min) was wider than that in Samples A, B, and C. However, more cross-links were formed between the protein molecules of Sample D than in the others (Samples A, B, and C). This is presumed to be the cause underlying the soft texture while maintaining the shape of the octopus muscles softened through vinegar pretreatment and sous vide softening treatment.

### 3.13. Acceptability Evaluation of Prototypes

Regarding the preference for appearance, Sample D had the highest score (7.8 points), but there was no significant difference when compared with that for Samples A, B, and C (*p* > 0.05). The preference for taste was confirmed to be higher in the order of Samples D (8.2 points), C (7.7 points), and B (7.5 points) compared to that for Sample A (raw), and there was no significant difference between Samples A, B, and C (*p* > 0.05). Regarding the preference for flavor, Samples B and D had the highest scores of 7.3 points and 7.8 points, respectively, whereas Sample A had the lowest score of 5.3 points, indicating a significant difference (*p* < 0.05). Regarding the preference for texture, Sample D scored the highest at 8.4 points (*p* < 0.05), while Sample B had the lowest score (4.3 points, *p* < 0.05).

## 4. Discussion

Response surface methodology (RSM) is a method for predicting an appropriate experimental model by identifying independent variables that can affect dependent variables and statistically analyzing the relationships between these independent variables [44,45] (Table 3). This is the most efficient method in terms of time and economy because it predicts the optimal condition of dependent variables using statistical techniques based on the experimental results obtained under specific conditions of the independent variable [46,47,48] (Table 4). In this study, the RSM technique was applied to efficiently develop Octopus pulpo as an elderly-friendly HMR to optimize the sous vide temperature, treatment time, and concentration of acetic acid. As shown in Table 5 and Table 6, and in Figure 1, the RSM design of the main manufacturing conditions for the elderly-friendly octopus pulpo HMR, predicted the following values: hardness (*Y_1_*) 405.0 × 1000 N/m^2^, total acidity according to sous vide softening treatment temperature (*X_1_*, 84.3°C), treatment time (*X_2_*, 139.8 min) and vinegar immersion concentration (*X_3_*, 0.48%) of octopus arms (total acidity, *Y_2_*) 550.7 mg/100 g, and sensory texture (*Y_3_*) of 4.8 points. When these predicted values were compared with the actual measured values of *Y_1_* 394.5 × 1000 N/m^2^, *Y_2_* 550.7 mg/100 g), and *Y_3_* 4.9 points, no significant difference was found, confirming that the RSM model was designed correctly.

In various studies, physical treatment techniques such as high-temperature and high-pressure treatment [49,50], superheated steam treatment [20,51,52,53,54,55], and ultra-high-pressure treatment [56,57] have been applied to alter the muscle tissue of seafood products. Further, the sous vide heat treatment method has been applied to aquatic product processing to meet various consumer needs [10,58,59,60,61,62]. Recently, Erdem et al. [10] studied the altered characteristics of cephalopods (octopus, cuttlefish, and squid) under sous vide cooking conditions; Xiao et al. [62] studied the altered characteristics of squid mantle muscles with sous vide, steaming, and boiling; and Wu et al. [61] studied the characteristics of scallop adductor muscles under sous vide cooking conditions. Although only a few studies have applied the sous vide method to octopus, it has been shown suitable for softening octopus [10]. However, the development of elderly-friendly foods using softened octopus and the sous vide method has not been reported. In this study, we attempted to optimize the sous vide softening and vinegar immersion conditions required for developing an elder-friendly octopus pulpo prototype.

Erdem et al. [10] studied the sous vide treatment of octopus (*Octopus vulgaris*) and found that the hardness of octopus cooked by sous vide (95 °C, 30 min) was 28.44 N, whereas that of raw octopus arm was 121.85 N, showing a hardness decrease of 76.6% and increased softening. However, in this study, the hardness (394.5 × 1000 N/m^2^) of octopus arms treated with vinegar immersion and sous vide was reduced by 83.0% compared to that of raw octopus arms (2323.7 × 1000 N/m^2^). These results confirm that the softening treatment method involving continuous vinegar immersion and sous vide treatment is effective for softening octopus arm tissue.

In other words, confirming the result value obtained using the RSM design, the sous vide softening treatment temperature and time showed a negative correlation with the octopus tissue hardness and a positive correlation with the descriptive analysis (seven-point scale method) score for the octopus tissue texture.

Upon confirming the texture item during the evaluation of the degree of preference by older consumers, the pulpo prototypes manufactured using only vinegar-treated octopus, only sous-vide-treated octopus, and both vinegar-immersion and sous-vide-treated octopus were evaluated; among these three prototypes, the pulpo prototype manufactured using octopus with both vinegar immersion and sous vide showed the highest overall acceptance (Table 12).

Compared to the preference score (4.3 points) for the octopus pulpo prototype prepared using raw octopus arms, the preference for the texture of the octopus pulpo prototype prepared using softened octopus arms among elderly consumers was 8.4 points (Table 12).

As shown in Figure 2, the cross-sections of raw octopus arm muscle (A), boiled octopus arm muscle (B), octopus arm muscle with only sous vide softening treatment (C), and octopus arm muscle after vinegar immersion (at the optimal RSM-derived concentration) and sous vide softening treatment (D) were compared by scanning electron microscopy. Microstructure observation (Figure 2) confirmed that the muscle fiber gaps in the arm muscles of the boiled octopus were widened (Figure 2B); in muscles treated only with sous vide softening, the muscle fiber spacing was further widened (Figure 2C), and the tissue was softened by cross-link formation between the protein molecules of the muscle that had undergone the sous vide softening process after vinegar immersion treatment (Figure 2D), compared to that in raw octopus arm muscles without any treatment (Figure 2A). According to a study by Wu et al. [61], when the microstructure of sous-vide-cooked scallop (*Argopecten irradians*), adductor muscles were observed, the myofibrils of sous-vide-cooked scallop adductor muscles at 65 °C (5.5 h) and 70 °C (1.5 h) were densely arranged. Further, the myofibrils of scallop adductor muscle-cooked sous vide at a higher temperature, 100 °C (5 min), were more cross-linked and aggregated. Wu et al. [61] reported that this change in the myofibril structure caused the softening of sous-vide-cooked scallop adductor muscle tissue. In this study, when the microstructure of the octopus arm muscles treated with sous vide softening was confirmed by electron microscopy (Figure 2), a trend similar to that reported by Wu et al. [61] was observed. Therefore, the softening of octopus muscle through sous vide treatment was assumed to be attributed to changes in myofibril structure caused by heat treatment. However, additional research is needed to elucidate the mechanism of muscle tissue softening in aquatic products by pretreatment with vinegar immersion.

Different from other studies, vinegar immersion treatment was performed before the sous vide softening treatment for octopus arms to prepare the octopus pulpo a La Gallega prototype (OPLGP). In general, vinegar contains organic acids, amino acids, and alcohols and is used for odor removal, tissue softening, flavor enhancement, and antibacterial activity during the processing of various foods [63,64,65]. The water content of the OPLGP prepared using the raw octopus arm (82.0 g/100 g) was greater than that of OPLGP prepared with octopus subjected to the optimal sous vide softening treatment temperature, treatment time, and vinegar immersion concentration (69.6 g/100 g), but the difference was not statistically significant (Table 7), presumably because the sous vide softening process inhibits water loss from the octopus arm muscles [10,66].

When the crude protein content (Table 7) and total amino acid content (Table 8) of OPLGP were compared, OPLGP showed an increase of 9.0% and 5.2%, respectively, compared with that in raw octopus arm muscle (*p* < 0.05). In particular, OPLPG added boiled potatoes (190 g; Table 1) to provide preference and nutritional functions for elderly consumers, so OPLPG’s methionine content (351.5 mg/100 g) was about twice as high as raw octopus arm’s methionine content (175.1 mg/100 g). That is, boiled potatoes have a low moisture loss rate, and the inside of the potatoes is heated quickly. In particular, the main scent and taste components of boiled potatoes are known to be caused by the Strecker decomposition of the Maillard reaction and methionine, so it was judged that it was most affected by methionine. These results can also confirm the supporting evidence in studies by Duckham et al. [67], Oruna-Concha et al. [68], and Jansky [69] that reported that sulfur amino acid, which has a high influence on heated potatoes, is methionine. This result is considered a positive factor for the intake of OPLGP by the elderly because it helps improve nutrition and health.

As shown in Table 9, the main mineral of OPLGP was potassium (170.6 mg/100 g), which was lower than that in raw octopus arm muscle (305.0 mg/100 g), followed by calcium, iron, and zinc. Lourenço et al. [70] and Santi et al. [71] reported that the main mineral in cephalopods, such as octopuses, is potassium (223.0 mg/100 g of raw common octopus, 277.5 mg/kg of raw cuttlefish). However, the mineral content of OPLGP was significantly lower than that of raw octopus arm muscle by 17.2%, 44.1%, and 50.0% for calcium, potassium, and zinc, respectively, but significantly higher for iron by 62.8% (*p* < 0.05).

As shown in Tables 10, 21, and 22, fatty acids (C14:0 to C22:6n-3) were identified in the fatty acid content of raw octopus arm muscle and OPLGP, respectively. The major fatty acids in raw octopus arm muscle were C22:6n-3 (EPA, 79.7 mg/100 g), C20:5n-3 (DHA, 54.2 mg/100 g), C16:0 (palmitic acid, 48.2 mg/100 g), and C18:1n-9 (oleic acid, 31.1 mg/100 g). However, the main fatty acids of OPLGP are C18:1n-9 (oleic acid, 3822.6 mg/100 g), C16:0 (palmitoleic acid, 856.8 mg/100 g), C18:2n-6 (linoleic acid, 665.1 mg/100 g), C22:6n-3 (EPA, 227.4 mg/100 g), and C20:5n-3 (DHA, 150.2 mg/100 g). Sioen et al. [72], when comparing fatty acid profiles of salmon (190 °C, 16 min) and cod (234 °C, 10 min) fried in olive oil; C18: 1n-9, C16: 0, C22: 6n-3, and C20: 5n-3 were higher in that order, showing results similar to the fatty acid profile of sous-vide-treated octopus in this study. Vegetable oils, such as olive oil and sunflower oil, are widely used for several purposes in the seafood processing industry and are known to contribute to the nutritional and organoleptic value of preserved foods [73]. In this study, we confirmed that the addition of olive oil for flavor enhancement and antibacterial activity had an effect similar to that of the fatty acid profile of olive oil [39,74].

As shown in Table 11, the OPLGP is an elderly-friendly HMR product that must be checked for safety when consumed by elderly consumers. Compared to octopus as a raw material, no hazards occurred during the manufacturing process in terms of pH, VBN, TBC, and heavy metals, which are indicators of freshness.

Table 12 shows the preference evaluation scores of octopus pulpo a La Gallega prepared with raw octopus arm muscle (A), boiled octopus arm muscle (B), octopus arm muscle softened only by sous vide (C), and octopus arm muscle that had undergone vinegar soaking and sous vide softening treatment (D) by elderly consumers (*n* = 30). The preference for the appearance of Samples A, B, C, and D was evaluated as similar, and there was no significant difference (*p* > 0.05). However, for taste, aroma, and texture (physical properties), Sample D showed a score difference of 2.1, 2.5, and 4.1, respectively, compared to Sample A (*p* < 0.05), with the preference for texture having the largest difference. This indicates that the texture of octopus arm muscle prepared by vinegar soak pretreatment and the sous vide softening process is the most suitable for consumption by elderly consumers. Further, Sample D was estimated to have the highest preference for taste and aroma because the off-flavor peculiar to octopus arm muscles was masked by the vinegar soak pretreatment [75,76]. Also, as in Samples D and C, the texture of the octopus arms treated with sous vide softening was superior to that of the arms without sous vide softening. This was presumed to be attributed to minimized moisture loss owing to the low water vapor permeability of the exclusive packaging used for the sous vide softening treatment [77]. Similarly, Humaid et al. [78] reported that sous-vide-treated lobster meat had a softer texture than without sous vide treatment, showing high acceptance.

## 5. Conclusions

In this study, the optimal manufacturing process for an HMR using vinegar-soaked and sous-vide-softened octopus arms was established using RSM. Further, after manufacturing octopus pulpo a La Gallega prototypes using softened octopus arms, their physical, chemical, microbiological, and sensory properties were confirmed. Overall, very-good-quality characteristics were confirmed, and “very likable” was the score in terms of overall acceptance, especially targeting elderly consumers. These results are expected to provide reference data for the development of various elder-friendly foods in the future and contribute to improving the quality of life of the elderly (especially those with masticatory disorders) in Korea.

## Figures and Tables

**Table 1:** Ingredients and contents for preparing octopus pulpo a La Gallega.

Ingredients		Contents (g)
Sous vide octopus arm	Octopus arm	756
	Vinegar	4
Shaped and boiled potato		190
Seasoning sauce	Olive oil	23
	Paprika powder	18
	Refined sugar	7
	Refined salt	1.5
	Black pepper	0.4
	Parsley	0.1
Total		1000

**Table 2:** Experimental range and values of independent variables in the central composite design for optimizing the manufacturing conditions of octopus pulpo a La Gallega.

Independent Variables		Symbol	Range Level				
			−1.682	−1	0	+1	+1.682
Sous vide treatment condition	Temp. (°C)	*X*_1_	70.0	76.0	85.0	94.0	100.0
	Time (min)	*X*_2_	69.5	90.0	120.0	150.0	170.5
Vinegar conc. (%)		*X*_3_	0.2	0.3	0.5	0.7	0.8

**Table 3:** Central composite design matrix and values of dependent variables for the sous vide treatment and vinegar soaking when preparing octopus pulpo a La Gallega.

Run Number		Independent Variables						Dependent Variables		
		Coded Values			Uncoded Values					
		*X*_1_	*X*_2_	*X*_3_	*X*_1_	*X*_2_	*X*_3_	*Y*_1_	*Y*_2_	*Y*_3_
Fractional factorial design (8 points)	1	−1	−1	−1	76.0	90.0	0.3	964.6	378.0	1.5
	2	+1	−1	−1	94.0	90.0	0.3	484.2	441.0	4.5
	3	−1	+1	−1	76.0	150.0	0.3	746.9	406.8	3.0
	4	+1	+1	−1	94.0	150.0	0.3	430.5	453.6	4.3
	5	−1	−1	+1	76.0	90.0	0.7	971.3	810.0	1.0
	6	+1	−1	+1	94.0	90.0	0.7	541.4	829.8	1.8
	7	−1	+1	+1	76.0	150.0	0.7	789.2	838.8	2.0
	8	+1	+1	+1	94.0	150.0	0.7	462.6	846.0	2.5
Axial portion (6 points)	9	−1.682	0	0	70.0	120.0	0.5	1081.8	594.0	1.2
	10	+1.682	0	0	100.0	120.0	0.5	524.8	646.2	3.5
	11	0	−1.682	0	85.0	69.5	0.5	672.0	612.0	3.5
	12	0	+1.682	0	85.0	170.5	0.5	454.5	682.0	4.2
	13	0	0	−1.682	85.0	120.0	0.2	559.9	267.8	3.8
	14	0	0	+1.682	85.0	120.0	0.8	651.4	919.8	1.0
Center points (3 points)	15	0	0	0	85.0	120.0	0.5	415.2	561.6	4.8
	16	0	0	0	85.0	120.0	0.5	395.1	550.8	4.8
	17	0	0	0	85.0	120.0	0.5	425.2	540.0	4.6

*X*_1_, Sous vide treatment temp. (°C); *X*_2_, Sous vide treatment time (min); *X*_3_, Vinegar conc. (%); *Y*_1_, Hardness (×1000 N/m^2^); *Y*_2_, Total acidity (mg/100 g); *Y*_3_, Sensory texture (points).

**Table 4:** Estimated coefficients of fitted quadratic polynomial equations for dependent variables based on the results of t-statistic.

Parameters	*Y*_1_		*Y*_2_		*Y*_3_	
	Coefficient	*p*-Value	Coefficient	*p*-Value	Coefficient	*p*-Value
Constant	411,728	0.000	550.74	0.000	4.7450	0.000
*X*_1_	−182,339	0.000	16.45	0.002	0.6933	0.000
*X*_2_	−65,759	0.000	14.95	0.003	0.3059	0.004
*X*_3_	21,406	0.013	200.76	0.000	−0.7841	0.000
*X*_1 × 1_	138,748	0.000	24.72	0.000	−0.8822	0.000
*X*_2_ *X*_2_	53,877	0.000	34.23	0.000	−0.3519	0.003
*X*_3_ *X*_3_	68,875	0.000	15.42	0.004	−0.8646	0.000
*X*_1_ *X*_2_	33,400	0.005	−3.60	0.431	−0.2500	0.032
*X*_1_ *X*_3_	5046	0.569	−10.35	0.047	−0.3750	0.005
*X*_2_ *X*_3_	1305	0.881	0.45	0.920	0.0500	0.609

*X*_1_, Sous vide treatment temp. (°C); *X*_2_, Sous vide treatment time (min); *X*_3_, Vinegar conc. (%); *Y*_1_, Hardness (×1000 N/m^2^); *Y*_2_, Total acidity (mg/100 g); *Y*_3_, Sensory texture (points).

**Table 5:** Optimization of the sous vide treatment and vinegar soaking for preparing octopus pulpo a La Gallega using RSM.

Dependent Variables	Values	Sous Vide Treatment Condition				Vinegar Soaking Condition	
		*X*_1_		*X*_2_		*X*_3_	
*Y*_1_	Target	400.0	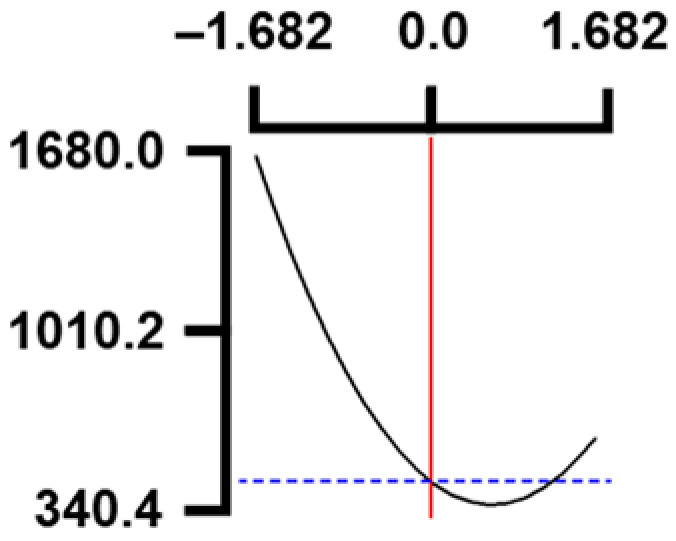	400.0	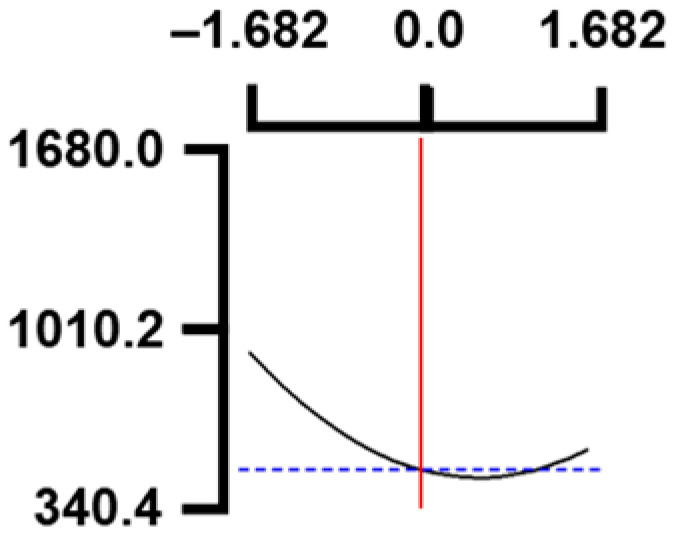	400.0	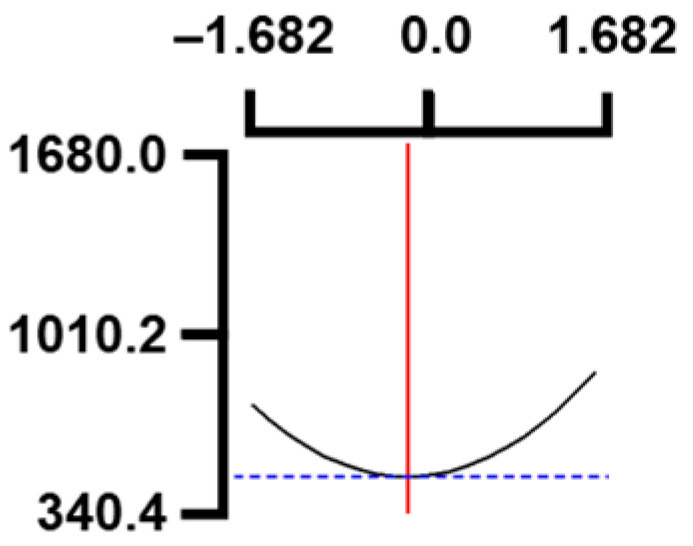
	Coded	0.00		0.00		−0.08	
	Actual	85.0		85.0		0.48	
*Y*_2_	Target	550.0	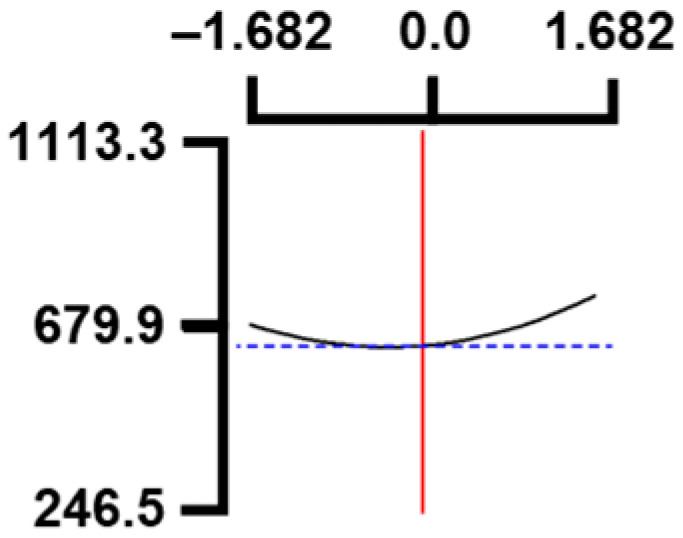	550.0	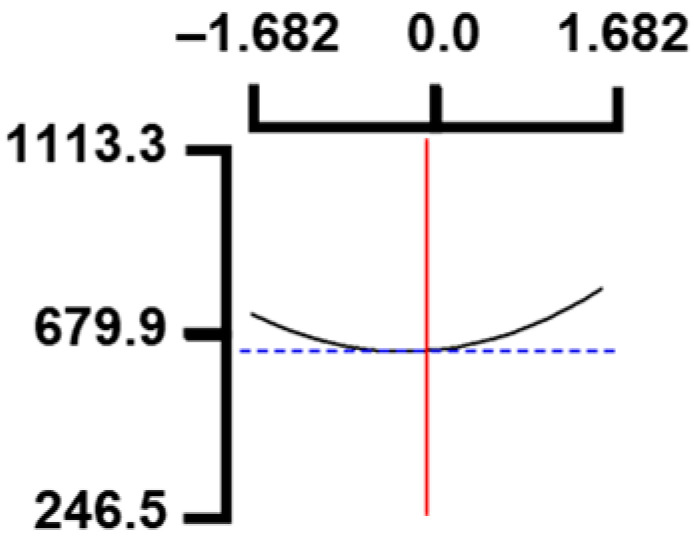	550.0	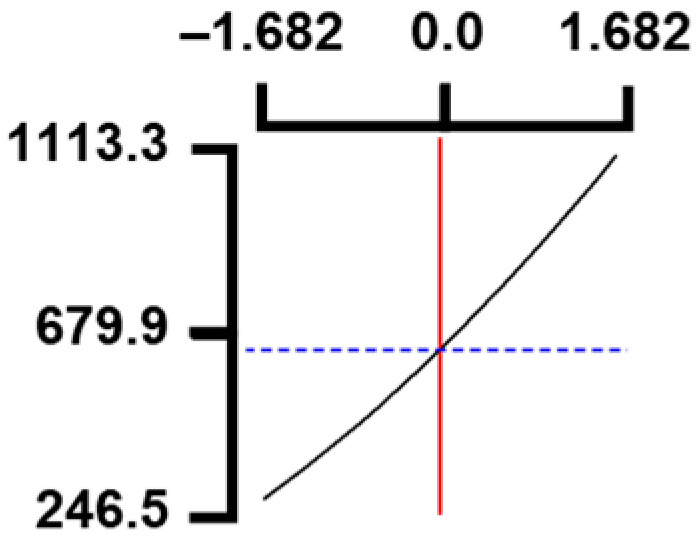
	Coded	0.00		0.00		0.00	
	Actual	85.0		85.0		0.5	
*Y*_3_	Target	Max.	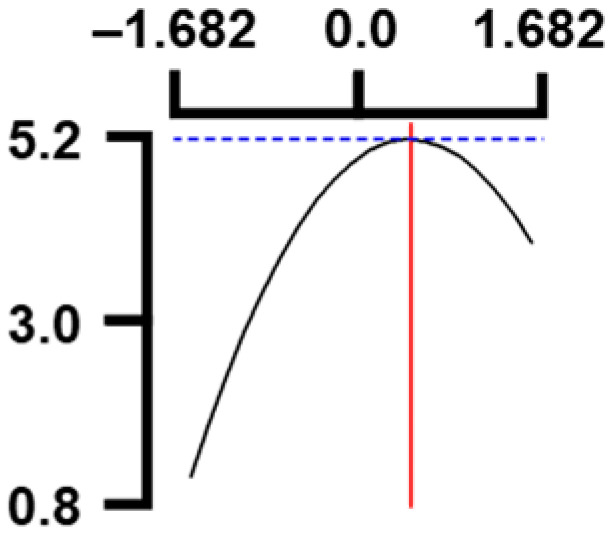	Max.	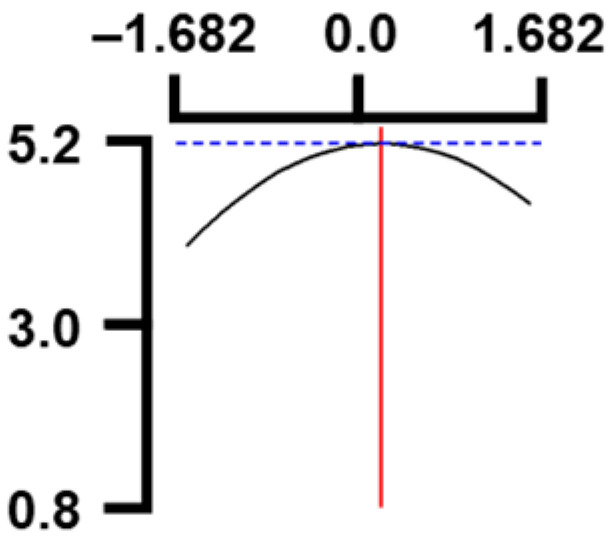	Max.	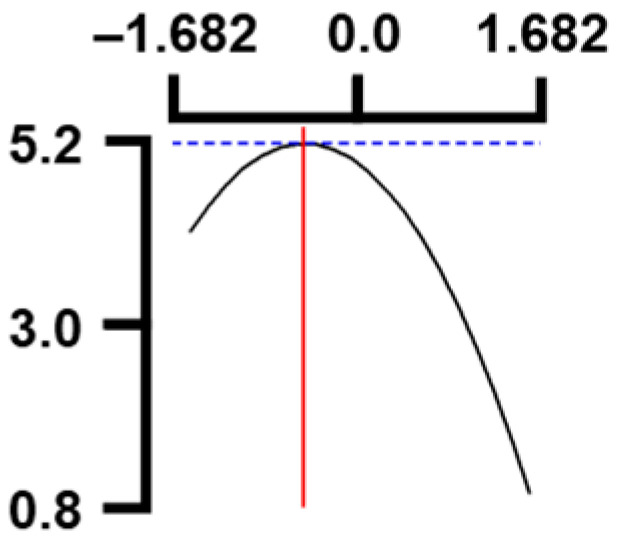
	Coded	0.49		0.49		−0.56	
	Actual	89.4		89.4		0.39	
Multiple response optimization	Coded	−0.08		0.66		−0.12	
	Actual	84.3		139.8		0.48	
	Predicted	*Y*_1_: 405.0 × 1000 N/m^2^, *Y*_2_: 550.7 mg/100 g, *Y*_3_: 4.8 score					

*X*_1_, Sous vide treatment temp. (°C); *X*_2_, Sous vide treatment time (min); *X*_3_, Vinegar conc. (%); *Y*_1_, Hardness (×1000 N/m^2^); *Y*_2_, Total acidity (mg/100 g); *Y*_3_, Sensory texture (points).

**Table 6:** Verification of predicted values for sous vide treatment and vinegar soaking for preparing octopus pulpo a La Gallega.

Dependent Variables	Predicted Values	Experimental Values
*Y*_1_ (Hardness, × 1000 N/m^2^)	405.0 ^a^	394.5 ± 13.0 ^a^
*Y*_2_ (Total acidity, mg/100 g)	550.7 ^a^	551.0 ± 0.8 ^a^
*Y*_3_ (Sensory texture, points)	4.8 ^a^	4.9 ± 0.1 ^a^

The letter a in the data indicate a significant difference at *p* > 0.05.

**Table 7:** Proximate composition, energy, and salinity contents of raw octopus arm and octopus pulpo a La Gallega prototype (OPLGP).

Sample		Raw	OPLGP
Proximate composition (g/100 g)	Moisture	82.0 ± 0.2 ^b^	69.6 ± 0.1 ^a^
	Crude protein	15.5 ± 0.1 ^a^	16.9 ± 0.1 ^b^
	Crude lipid	0.6 ± 0.0 ^a^	7.9 ± 0.0 ^b^
	Ash	1.7 ± 0.1 ^a^	1.8 ± 0.0 ^a^
	Carbohydrate	0.2	3.8
Energy (kcal)		72.4	142.4
Salinity (g/100 g)		0.6 ± 0.0 ^a^	0.6 ± 0.0 ^a^

Carbohydrate (%) = 100 − (moisture + crude protein + crude lipid + ash); Energy (kcal/100 g) = (Crude protein × 4.27) + (Crude lipid × 9.02) + (Carbohydrate × 3.87); Different letters on the data in the column indicate a significant difference at *p* < 0.05.

**Table 8:** Total amino acid contents (mg/100 g) and ratios (%) in raw octopus arm and octopus pulpo a La Gallega prototype (OPLGP).

EAA	Raw	OPLGP	NEAA	Raw	OPLGP
Threonine	706.2 (4.7)	772.9 (4.9)	Aspartic acid	1576.1 (10.6)	1737.7 (11.1)
Valine	686.0 (4.6)	659.9 (4.2)	Serine	694.1 (4.7)	784.5 (5.0)
Methionine	175.1 (1.2)	351.5 (2.3)	Glutamic acid	2384.3 (16.0)	2500.8 (16.0)
Isoleucine	744.6 (5.0)	725.8 (4.6)	Proline	677.7 (4.5)	769.9 (4.9)
Leucine	1189.7 (8.0)	1275.5 (8.2)	Glycine	950.3 (6.4)	994.9 (6.4)
Phenylalanine	949.3 (6.4)	721.3 (4.6)	Alanine	830.3 (5.6)	866.8 (5.5)
Histidine	340.9 (2.3)	372.1 (2.4)	Cysteine	69.0 (0.5)	119.1 (0.8)
Lysine	1196.6 (8.1)	1221.5 (7.8)	Tyrosine	411.9 (2.8)	533.4 (3.4)
Arginine	1275.4 (8.6)	1224.8 (7.8)	Sub-total	7593.7 (51.1)	8307.1 (53.1)
Tryptophan	-	-	Total	14,857.5 (100.0)	15,632.4 (99.9)
Sub-total	7263.8 (48.9)	7325.3 (46.8)			

EAA, Essential amino acid; NEAA, Non-essential amino acid. The value of parenthesis means a percentage of each amino acid content to total amino acid content.

**Table 9:** Mineral contents (mg/100 g) of raw octopus arm and octopus pulpo a La Gallega prototype (OPLGP).

Mineral Contents	Raw	OPLGP
Calcium (Ca)	26.2 ± 0.2 ^b^	21.7 ± 0.3 ^a^
Potassium (K)	305.0 ± 3.1 ^b^	170.6 ± 4.0 ^a^
Iron (Fe)	3.5 ± 0.0 ^a^	5.7 ± 0.0 ^b^
Zinc (Zn)	0.8 ± 0.0 ^b^	0.4 ± 0.0 ^a^

Different letters on the data in the column indicate a significant difference at *p* < 0.05.

**Table 10:** Fatty acid contents (mg/100 g) and ratio (%) in raw octopus arm and octopus pulpo a La Gallega prototype (OPLGP).

FA	Raw	OPLGP	FA	Raw	OPLGP	FA	Raw	OPLGP
C12:0	ND	ND	C14:1	0.1 (0.0)	ND	C20:3n-6	0.2 (0.1)	2.0 (0.0)
C14:0	3.6 (1.3)	13.0 (0.2)	C16:1	5.8 (2.0)	68.4 (1.1)	C20:3n-3	1.0 (0.4)	6.3 (0.1)
C15:0	0.6 (0.2)	3.8 (0.1)	C18:1n-9	31.1 (10.7)	3822.6 (60.6)	C22:2NMID	0.5 (0.2)	6.9 (0.1)
C16:0	48.2 (16.7)	856.8 (13.6)	C20:1n-9	12.8 (4.4)	38.9 (0.6)	C20:5n-3	54.2 (18.7)	150.2 (2.4)
C17:0	4.8 (1.7)	23.2 (0.4)	C22:1n-9	0.9 (0.3)	18.9 (0.3)	C22:6n-3	79.7 (27.5)	227.4 (3.6)
C18:0	19.5 (6.7)	245.9 (3.9)	C24:1n-9	0.3 (0.1)	ND	ƩPUFA	144.6 (50.0)	1111.5 (17.6)
C20:0	0.2 (0.1)	28.1 (0.4)	ƩMUFA	51.0 (17.5)	3948.8 (62.6)	Ʃω-6	7.2 (2.5)	667.1 (10.6)
C23:0	16.7 (5.8)	57.4 (0.9)	C18:2n-6	7.0 (2.4)	665.1 (10.5)	Ʃω-3	135.7 (46.9)	437.5 (6.9)
C24:0	0.2 (0.1)	16.6 (0.3)	C18:3n-3	0.8 (0.3)	53.6 (0.9)	Total FA	289.4 (100.1)	6305.1 (99.9)
ƩSFA	93.8 (32.6)	1244.8 (19.7)	C20:2NMID	1.2 (0.4)	ND	Total Lipid	600 (48.2)	7900 (79.8)

FA, Fatty acid; ND, Not detected; 20:5n-3, Docosahexaenoic acid (DHA); 22:6n-3, Eicosapentaenoic acid (EPA); ƩSFA, Saturated fatty acid; ƩMUFA, Monounsaturated fatty acid; ƩPUFA, Polyunsaturated fatty acid. The value in parentheses is the ratio (%) of each fatty acid to the total fatty acid.

**Table 11:** The pH, volatile basic nitrogen (VBN), total bacterial counts (TBC), and heavy metal contents of raw octopus arm and octopus pulpo a La Gallega prototype (OPLGP).

Sample		Raw	OPLGP
Freshness indicators	pH	6.70 ± 0.0 ^a^	6.92 ± 0.0 ^b^
	VBN (mg/100 g)	6.5 ± 1.6 ^a^	14.8 ± 0.8 ^b^
	TBC (CFU/g)	2.0 × 10 ^b^	1.2 × 10 ^a^
Heavy metal	Pb (mg/kg)	0.06 ± 0.00 ^b^	0.01 ± 0.00 ^a^
	Cd (mg/kg)	0.09 ± 0.00 ^b^	0.02 ± 0.00 ^a^

Different letters on the data in the column indicate a significant difference at *p* < 0.05.

**Table 12:** Acceptability evaluation of the octopus pulpo a La Gallega prototype manufactured using octopus arm (A, Raw octopus arm; B, Boiled octopus arm; C, Sous vide octopus arm; D, Soaked in vinegar and sous vide octopus arm).

Acceptability Evaluation	A (Raw)	B (Boiled)	C (Sous Vide)	D (Vinegar/ Sous Vide)
Appearance	6.8 ± 0.9 ^a^	7.1 ± 0.9 ^a^	7.4 ± 0.7 ^a^	7.8 ± 0.6 ^a^
Taste	6.1 ± 0.6 ^a^	7.5 ± 0.7 ^b^	7.7 ± 0.5 ^b^	8.2 ± 0.6 ^b^
Flavor	5.3 ± 0.7 ^a^	7.3 ± 1.0 ^b^	7.2 ± 0.6 ^b^	7.8 ± 0.7 ^b^
Texture	4.3 ± 0.7 ^a^	6.3 ± 0.9 ^b^	7.4 ± 0.9 ^b^	8.4 ± 0.9 ^b^
Overall acceptance	5.6 ± 0.3 ^a^	7.0 ± 0.5 ^b^	7.4 ± 0.4 ^b^	8.0 ± 0.4 ^b^

Panel configurations, *n* = 30 (15 males and 15 females); the average age, 70 (65–75); Mean ± S.D; Different letters on the data in the column indicate a significant difference at *p* < 0.05.

## Data Availability

The data used to support the findings of this study can be made available by the corresponding author upon request.

## Supplementary Materials

The following supporting information can be downloaded at: https://www.mdpi.com/article/10.3390/foods12183343/s1, Figure S1: Operation method of the texture property meter in level 1 of texture.
