# Supplementary material for: Development and Quality Characteristics of Elderly-Friendly Pulpo a La Gallega Prepared Using Texture-Modified Octopus (Octopus vulgaris) Arms
Source: Foods. 2023 Sep 6;12(18):3343. doi: 10.3390/foods12183343 (PMC10527877; doi:10.3390/foods12183343)
Supplement: Supplementary file 1 [file foods-12-03343-s001.zip › foods-2573736-supplementary.pdf]

foods

MDPI

Supplementary material

Development and Quality Characteristics of Elderly-Friendly Pulpo a La Gallega Prepared Using Texture-Modified Octopus (*Octopus vulgaris*) Arms

Sang-In Kang[1], Jin-Soo Kim[2], Sun-Young Park[2], Seok-Min Lee[2], Mi-Soon Jang[3], Jae-Young Oh[3] and Jae-Suk Choi[2,*]

[1] Seafood Research Center, IACF, Silla University, 606, Advanced Seafood Processing Complex, Wontang=ro, Amanam-dong, Seo-gu, Busan, 49277, Republic of Korea; ftrnd5@silla.ac.kr

[2] Department of Seafood Science and Technology, Institute of Marine Industry, Gyeongsang National University, 2-9, Tongyeonghaean-ro, Tonyeong-si, Gyeongsangnam-do, 53064, Republic of Korea; jinsukim@gnu.ac.kr (J.-S.K.); tjsdud3591@gnu.ac.kr (S.Y.P.); tjrals03001@gnu.ac.kr (S.M.L.)

[3] Food Safety and Processing Research Division, National Institute of Fisheries Science, 216, Gijanghaean-ro, Gijang-eup, Busan 46083, Republic of Korea; suni1@korea.kr (M.-S.J.); ojy0724@korea.kr (J.-Y.O.)

* Correspondence: jsc1008@gnu.ac.kr; Tel.: +82-55-772-9142

Experiment method 1

Grade 1 (tooth-enabled intake) senior-friendly food is a regular product; therefore, in the case of a sample for measuring its hardness, a sample with a size of 20 mm or more and wider than the area of the probe was used without any pretreatment like cutting, if possible. "Experiment method 1" for hardness measurement conditions using a rheometer was as follows. A compression speed of 100 mm/min and a sample temperature of 20 ± 2 °C were set using a circular probe with a diameter of 5 mm and an acrylic bottom plate for measuring the physical properties (thickness, 10 mm; center hole diameter, 10 mm). The depth was measured by completely penetrating the test product (Figure S1).

Experiment method 3

"Experiment method 3" for hardness measurement conditions using a rheometer was as follows. A compression rate of 600 mm/min and a sample temperature of 20 ± 2°C were set using a circular probe with a diameter of 3 mm, and clearance was measured five times up to 30% of the sample thickness. Among the results obtained from five measurements, the average value of three measurements, excluding the maximum and minimum values, is shown.

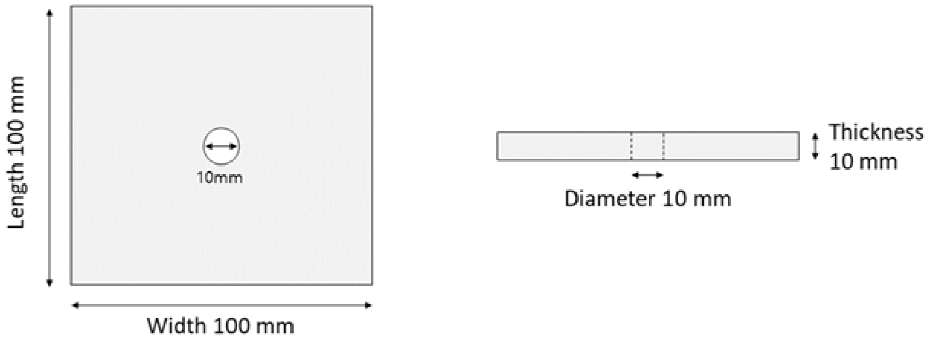

Plate for measurement of texture properties

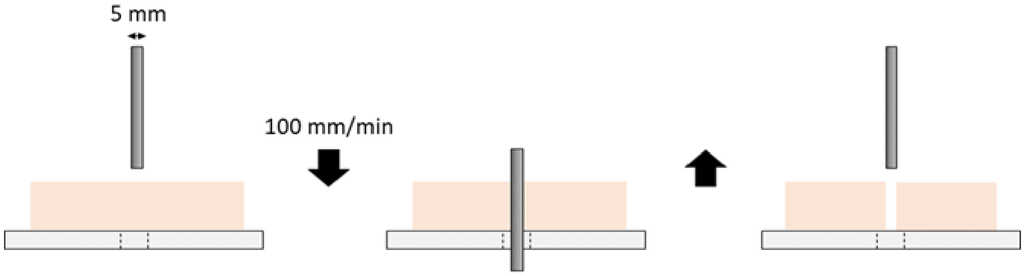

Operation in accordance with the "experiment method 3"

Figure S1. Operation method of the texture property meter in level 1 of texture.
